# Risk Factors and Outcome of Acute Kidney Injury after Isolated CABG Surgery: a Prospective Cohort Study

**DOI:** 10.21470/1678-9741-2017-0209

**Published:** 2019

**Authors:** Shahram Amini, Mona Najaf Najafi, Seyedeh Parissa Karrari, Mohammadghasem Etemadi Mashhadi, Sahereh Mirzaei, Mohammad Abbasi Tashnizi, Ali Asghar Moeinipour, Hamid Hoseinikhah, Mathias Hossain Aazami, Mahdieh Jafari

**Affiliations:** 1 Department of Anesthesiology and Critical Care, Mashhad University of Medical Sciences, Mashhad, Iran.; 2 Clinical Research Unit, Faculty of Medicine, Mashhad University of Medical Sciences, Mashhad, Iran.; 3 Department of Anesthesia, Faculty of Medicine, Mashhad University of Medical Sciences, Mashhad, Iran.; 4 Department of Biobehavioral Sciences, College of Nursing, University of Illinois at Chicago, Chicago, USA.; 5 Department of Cardiac Surgery, Mashhad University of Medical Sciences, Mashhad, Iran.

**Keywords:** Acute Kidney Injury, Coronary Artery Bypass, Treatment Outcome

## Abstract

**Background:**

Acute kidney injury (AKI) is a frequent event after cardiac surgery with increased mortality and morbidity. We explored frequency, risk factors, and associated morbidity and mortality of AKI after isolated coronary artery bypass grafting (CABG) surgery at a single institution.

**Methods:**

All consecutive adults undergoing CABG surgery from March 2013 to October 2016 were assessed for development and severity of AKI based on Acute Kidney Injury Network (AKIN) criteria. The patients were also investigated regarding their need for renal replacement therapy (RRT), predictive risk factors, and associated outcomes, including duration of mechanical ventilation, mortality, intensive care unit (ICU) and hospital length of stay.

**Results:**

Of 1737 patients in the study, 275 (15.8%) developed AKI. Twenty-five (12.8%) cases required RRT. Patients with AKI had longer ventilation time, ICU and hospital length of stay (*P*<0.001). Mortality rates were 28 (10.2%) and 22 (1.5%) in patients with and without AKI, respectively (*P*<0.001). There was a strong association between advanced age (aOR=1.016, 95% CI=1.002-1.030, *P*=0.028), diabetes (aOR=1.36, 95% CI=1.022-1.809, *P*=0.035), on-pump surgery (aOR=2.63, 95% CI=1.543-4.483, *P*<0.001), transfusion of more than 1 unit of red blood cells (aOR=2.154, 95% CI=1.237-3.753, *P*=0.007), and prolonged mechanical ventilation and development of AKI (aOR=2.697, 95% CI=1.02407.071, *P*<0.001). AKI was seen less frequently in those with opium abuse (aOR=0.613, 95% CI=0.409-0.921, *P*=0.018).

**Conclusion:**

We demonstrated that advanced age, diabetes, on-pump surgery, red blood cell transfusion, and prolonged mechanical ventilation were independent positive risk factors for the development of AKI after isolated CABG while opium abuse was a protective factor.

**Table:** 

Abbreviations, acronyms & symbols	
AKI	= Acute kidney injury
AKIN	= Acute Kidney Injury Network
CABG	= Coronary artery bypass grafting
CPB	= Cardiopulmonary bypass
eGFR	= Estimated glomerular filtration rate
IABP	= Intra-aortic balloon pump
ICU	= Intensive care unit
RBC	= Red blood cells
ROC	= Receiver operating characteristic
RRT	= Renal replacement therapy

## INTRODUCTION

Acute kidney injury (AKI) is a common complication after cardiac surgery. The incidence has been reported from 6.7% to 39%^[1-4]^, based on definition of AKI. It is associated with increased morbidity and mortality that is further increased in more severe stages of AKI^[5-7]^. It also increases intensive care unit (ICU) and hospital length of stay^[2,8]^, and use of resources^[2]^. Renal replacement therapy (RRT) is required in 1.6% to 7.7% after cardiac surgery, which may further increase the mortality rate in these patients^[9,10]^.

Several perioperative risk factors have been reported as predictors of AKI associated with cardiac surgery, including advanced age^[11,12]^, time interval of angiography and surgery^[13]^, blood transfusion^[4,11,14]^, preoperative elevated serum creatinine^[15,16]^, diabetes^[17]^, high-risk according to the EuroSCORE^[18]^, and use of nephrotoxic drugs. Use of cardiopulmonary bypass (CPB) has also been proposed to be a predictive factor for development of AKI after coronary artery bypass grafting (CABG)^[19,20]^. In addition to the use of prophylactic drugs^[21]^, knowing the predisposing factors and modifying them can help to decrease the incidence of AKI.

The objective of this study was to explore the frequency of AKI after isolated CABG at a single teaching hospital. The primary outcome was to determine the frequency of AKI. The secondary outcomes included determination of risk factors and associated outcomes, including duration of mechanical ventilation, ICU and hospital length of stay, and in-hospital mortality.

## METHODS

After obtaining approval from the university's ethics committee, we recruited all consecutive adults undergoing isolated CABG from March 2013 to October 2016 at a university teaching hospital in Mashhad, Iran. Patients were excluded if they had no documented preoperative serum creatinine or expired within 24 hours.

Baseline variables included demographic parameters, preoperative hemoglobin, urea, creatinine, creatinine clearance, and any comorbidities.

Surgical characteristics including type of surgery, duration of surgery, use of CPB and intraoperative events, including excessive bleeding, life-threatening arrhythmia, and intraoperative transfusion were recorded.

All patients underwent standard anesthesia and were transferred to cardiac surgery ICU where they were under standard monitoring and meticulous attention by intensive care team including intensivists, critical care fellows, anesthesiology residents, and trained registered nurses to maintain optimal cardiopulmonary, cerebral and renal function.

The patients were assessed postoperatively for development, severity, time of onset, and duration of AKI. Urine output was not used as a criterion of AKI in our study due to the frequent use of diuretics in these patients. AKI was defined based on the Acute Kidney Injury Network (AKIN) criteria (Table 1). Estimated glomerular filtration rate (eGFR) was calculated using Cockroft-Gault equation. RRT was used in the case of refractory acid-base and electrolyte disorders, signs of hypervolemia or uremic encephalopathy (defined as decreased level of consciousness, assuming to be related to hyperuremia).

**Table 1:** AKIN criteria.

Serum creatinine criteria	Urine output criteria
Definition	
Increase in SCr by 0.3 mg/dL or ≥1.5 times baseline within 48 hours	
Staging	
1: Increase in SCr by ≥ 0.3 mg/dL or ≥1.5-<2.0 times baseline	<0.5 mL/kg per hour for more than 6 hours
2: Increase in SCr by 2.0-<3.0 times baseline	<0.5 mL/kg per hour for more than 12 hours
3: Increase in SCr by ≥3.0 times baseline	<0.3 mL/kg per hour for 24 hours or anuria for 12 hours

SCr=serum creatinine

They were also investigated for mechanical ventilation for more than 24 hours, ICU and hospital length of stay, and in-hospital death. Patients were weaned from mechanical ventilation and were extubated according to a standardized protocol.

### Statistical Analysis

Means and standard deviation were used for normal distribution variables and median and interquartile range for otherwise. Frequencies and percentages were used for categorical variables.

The Student's *t*-test or the Mann-Whitney test was used to compare continuous variables between patients with and without AKI. Chi-square test or Fisher's exact test was used for categorical variables.

Binary logistic regression model was constructed for assessment of independent effect of each variable on predicting the occurrence of AKI. Model goodness of fit was evaluated by the Hosmer-Lemeshow test. The receiver operating characteristic (ROC) curve was used for prediction of sensitivity and specificity of cutoff point of significant variables. Statistical significance was considered as *P* value of <0.05. The statistical analysis was performed using SPSS(r) version 16 (IBM SPSS, Chicago, IL, USA).

## RESULTS

During the study period, 1737 patients were recruited. CPB was used in 293 (16.9%) cases. There were 1073 (61.8%) males and 664 (38.2%) females, with a mean age of 59.93 ± 10.34 years. Demographic data and surgical characteristics of patients are presented in Table 2.

**Table 2:** Demographic data and patients' characteristics.

	Total	AKI	No AKI	P-value
Age (years)	60 (53-67)	61 (55-70)	59 (53-66)	0.001
Sex (male)	1073 (61.8)	167 (60.7)	906 (62)	0.74
Weight (kg)	70 (62-79)	71 (63-79)	70 (61-79)	0.46
Height (cm)	165 (158-171)	165 (158-170)	165 (158-171)	0.86
BMI	25.71 (23.24-28.72)	25.71 (23.36-29.16)	25.70 (23.24-28.66)	0.37
MI	152 (8.8)	24 (8.7)	128 (8.8)	>0.99
HTN	829 (47.7)	140 (50.9)	689 (47.1)	0.26
Diabetes	592 (34.1)	106 (38.5)	486 (33.2)	0.09
HLP	551 (31.7)	90 (32.7)	461 (31.5)	0.72
EF	50 (40-55)	50 (40-55)	50 (40-55)	0.17
NYHA (I/II/III/IV)	248/951/492/46	33/148/85/9	215/803/407/37	0.12
Left main or 3VD	1117 (64.3)	182 (66.2)	935 (64)	0.49
Smoking	223 (12.8)	29 (10.5)	194 (13.3)	0.24
Opium abuse	335 (19.3)	38 (13.8)	297 (20.3)	0.012
Baseline creatinine	1 (0.9-1.2)	1 (0.9-1.3)	1 (0.9-1.2)	0.27
Baseline GFR	70.10 (54.84-87.55)	66.70 (47.01-86.73)	70.42 (56.09-88.06)	0.009
Date of angiography	12 (4-24)	11 (3-26)	12 (5-24)	0.34
Use of IABP	26 (1.5)	8 (2.9)	18 (1.2)	0.053
Transfusion of red blood cells	73 (4.2)	26 (9.5)	47 (3.2)	<0.001

Data are presented as median ± IQR (interquartile range) or numbers (percentage). 3VD=3 vessel disease; AKI = Acute kidney injury; BMI=body mass index; EF=ejection fraction; GFR=glomerular filtration rate; HLP=hyperlipidemia; HTN=hypertension; IABP=intra-aortic balloon pump; LM=left main; MI=myocardial infarction; NYHA=New York Heart Association

AKI was seen in 275 (15.8%) patients. Of these, 81.8%, 8.4% and 9.8% cases developed stage I, II, and III, respectively. The median duration of AKI was 2 days. One hundred and eighty-one (65.8%) patients recovered with a median duration of 1 day. In 94 (34.2%) patients, AKI lasted during their hospital stay and 25 (12.8%) patients required RRT.

AKI was seen in 193 (13.4%) and 82 (28%) cases in patients with and without CPB, respectively (*P*<0.001), with the highest frequency in those undergoing on-pump beating surgery (Table 3).

**Table 3:** Frequency and stages of acute kidney injury in different surgical techniques.

	Off-pump	On-pump	On-pump beating	*P*-value
Total	1444 (83.1)	204 (11.7)	89 (1.5)	
No AKI	1251 (86.6)	153 (75)	58 (65.2)	<0.001
AKI	193 (13.4)	51 (25.0)	31 (34.8)	
AKIN 1	159 (82.4)	40 (78.4)	26 (83.9)	
AKIN 2	15 (7.8)	6 (11.8)	2 (6.5)	
AKIN 3	19 (9.8)	5 (9.8)	3 (9.7)	
RRT	13 (0.9)	8 (3.9)	4 (4.5)	

AKI=Acute kidney injury; AKIN=Acute kidney injury network

Mortality was seen in 28 (10.2%) and 22 (1.5%) patients with and without AKI, respectively (*P*<0.001), with 18 (8%), 3 (13%) and 7 (25.9%) cases in those with stage I, II, and III, respectively.

The median duration of mechanical ventilation was 320 minutes (222.5-502.5), with 360 (240-675) and 315 (220-480) minutes in those with and without AKI, respectively (*P*< 0.001).

Prolonged ventilation time (defined as ventilation over 24 hours) occurred in 11 (4%) and 15 (1%) patients with and without AKI, respectively (*P*<0.001). Duration of ICU stay was 2^[2-4]^ days in patients with AKI and 2 (2-3) days in those without AKI (*P*< 0.001). Hospital length of stay was 7 (6-11) and 7 (6-8) days in patients with and without AKI, respectively *(P*<0.001).

There was an association between advanced age, drug abuse, type of surgery, use of CPB, surgical duration, use of intra-aortic balloon pump (IABP), transfusion of red blood cells (RBC), and prolonged mechanical ventilation and development of AKI (Table 4). Multivariate regression analysis showed that advanced age, diabetes, use of CPB, transfusion of RBC, and prolonged mechanical ventilation were significantly associated with AKI (Table 5). In addition, opium abuse was a protective negative factor (Table 5).

**Table 4:** Univariate analysis of risk factors associated with acute kidney injury after coronary artery bypass grafting.

	Β	OR	95% CI	*P*-value
Preoperative				
Age (years)	0.021	1.02	1.008-1.03	<0.001
Sex (male)	-0.052	0.949	0.729-1.24	0.69
Diabetes	0.231	1.26	0.965-1.644	0.089
Drug abuse	-0.464	0.629	0.436-0.906	0.013
Baseline GFR	-0.005	0.995	0.990-1.001	0.08
Intraoperative				
Surgery type (ref.: off-pump):				
On-pump	0.77	2.161	1.521-3.07	<0.001
On-pump beating	1.243	3.464	2.183-5.497	<0.001
Duration of surgery	0.003	1.003	1.001-1.005	0.002
Use of IABP	0.877	2.404	1.035-5.584	0.041
Postoperative				
Transfusion of red blood cells	1.145	3.144	1.911-5.171	<0.001
Mechanical ventilation >24 hours	1.391	4.019	1.826-8.848	0.001

GFR=glomerular filtration rate; IABP=Intra-aortic balloon pump

**Table 5:** Multivariate analysis of risk factors associated with acute kidney injury after coronary artery bypass grafting.

	Β	OR	95% CI	*P*-value
Preoperative				
Age (years)	0.016	1.016	1.002-1.030	0.028
Diabetes	0.308	1.360	1.022-1.809	0.035
Opium abuse	-0.489	0.613	0.409-0.921	0.018
Intraoperative				
Surgery type (ref.: off-pump):				
On-pump	0.651	1.917	1.307-2.811	0.001
On-pump beating	0.967	2.63	1.543-4.483	<0.001
Transfusion of red blood cells	0.768	2.154	1.237-3.753	0.007
Postoperative				
Mechanical ventilation >24 hours	0.99	2.691	1.024-7.071	<0.001

OR=adjusted odds ratio; 95% CI=95% confidence interval

## DISCUSSION

Our study revealed that AKI is a common finding after isolated CABG with increased mortality and morbidity, including duration of mechanical ventilation, ICU and hospital length of stay. We also demonstrated that advanced age, diabetes, on-pump surgery, transfusion of RBC, and prolonged mechanical ventilation were independent positive risk factors for development of AKI, while drug abuse was a protective factor.

A number of mechanisms have been proposed as the etiology for development of AKI after heart surgery, including altered renal blood flow, hypoperfusion, inflammation, loss of pulsatile flow, ischemia, decreased autoregulation and nephrotoxic medications^[9]^.

Our findings are in accordance with previous studies demonstrating that advanced age, diabetes, use of CPB and transfusion are independent positive risk factors for AKI.

Similar to a review by Karkouti^[14]^, we found that transfusion of RBC was associated with an increased rate of AKI (OR=2.154). Decreased RBC deformability leading to small capillary obstruction, inability to handle oxygen due to deficient 2, 3-diphosphoglycerate and short lifespan of transfused RBC resulting in hemolysis and production of more free iron are the proposed mechanism of AKI associated with transfusion. However, in another study Karkouti et al.^[22]^ reported that this effect had been positive only in anemic patients.

We could not find an association between time interval of angiography and surgery and AKI. This is similar to findings of Ko et al.^[23]^, but in contrast to Hu et al.^[13]^. It seems that only a close succession of angiography and surgery can be associated with higher incidence of AKI after CABG. In our study, the median time interval between angiography and surgery was 12days^[4-24]^.

We demonstrated a strong association between AKI and advanced age. However, the association does not seem to be clinically significant (OR=1.016). This can be attributed to the younger age of our patients. Similar findings were reported by Ried et al.^[12]^.

There is no agreement on association of CPB and postoperative AKI. While Schopka et al.^[24]^ reported no protective effect of off-pump *versus* on-pump surgery, a meta-analysis by Seabra et al.^[19]^ revealed that patients undergoing off-pump CABG were 40% less affected by AKI than on-pump CABG. We found that use of CPB was strongly associated with AKI with on-pump beating technique as the most common causative factor. It was not possible to perform a statistical analysis on frequency of different stages of AKI and the need for RRT among patients undergoing different surgical techniques due to their low frequency. However, there does not seem to be a significant clinical difference regarding frequency of severe AKI (defined as stage 3 of AKIN) and need for RRT among the three groups.

In line with Oezkur et al.^[17]^, we also noted that diabetes was an independent risk factor for AKI. Interestingly, Kocogulları et al.^[25]^ suggested an association between preoperative level of Hb A_1C_ and the development of AKI in non-diabetics.

AKI was seen less commonly in patients using opium in our study. Hence, opium abuse might have a protective effect against AKI (OR=0.613, 95% CI=0.409-0.921). This can be attributed to possible antioxidant properties of natural opium used in our area. However, this cannot be extrapolated to synthetic opioids. As far as we know, this is the first study demonstrating such an effect and larger multicenter studies are needed to confirm this finding.

Our study had a few limitations. First, we did not investigate the changes between preoperative and intraoperative blood pressure that might affect the incidence of AKI^[26]^. Second, we did not use urine output as a definition for AKI since many patients received diuretics and this may affect accuracy of urine output as a variable for definition of AKI. Finally, the sample size for different techniques of CABG was not similar due to the preference and experience of different surgeons that may affect the results.

## CONCLUSION

In conclusion, we found that CABG associated AKI increases mortality and morbidity. Furthermore, advanced age, diabetes, use of CPB, transfusion of RBC, and prolonged mechanical ventilation are independent risk factors for the development of AKI, while drug abuse is a negative risk factor.

**Table:** 

Authors' roles & responsibilities	
SA	Substantial contributions to the conception or design of the work; or the acquisition, analysis, or interpretation of data for the work; drafting the work or revising it critically for important intellectual content; agreement to be accountable for all aspects of the work in ensuring that questions related to the accuracy or integrity of any part of the work are appropriately investigated and resolved; final approval of the version to be published
MNN	Analysis and interpretation of data for the work; final approval of the version to be published
SPK	Acquisition of data for the work; final approval of the version to be published
MEM	Acquisition of data for the work; final approval of the version to be published
SM	Acquisition of data for the work; final approval of the version to be published
MAT	Substantial contributions to the conception or design of the work; final approval of the version to be published
AAM	Substantial contributions to the conception or design of the work; final approval of the version to be published
HH	Substantial contributions to the conception or design of the work; final approval of the version to be published
MHA	Substantial contributions to the conception or design of the work; final approval of the version to be published
MJ	Acquisition of data for the work; final approval of the version to be published
